# Qualitative perspectives of Medicaid-insured patients on ambulatory care at an academic medical center: challenges and opportunities

**DOI:** 10.1186/s12913-024-11619-3

**Published:** 2024-09-27

**Authors:** Mariah B. Blegen, Jessica Faiz, Daniel Gonzalez, Vanessa Nuñez, Nina Harawa, Medell Briggs-Malonson, Gery Ryan, Katherine L. Kahn

**Affiliations:** 1 VHA HSR Center for the Study of Healthcare Innovation, Implementation, and Policy (CSHIIP), Greater Los Angeles VA Medical Center, Los Angeles, CA USA; 2 Department of Surgery, David Geffen School of Medicine at UCLA, 10833 Le Conte Ave. 72-227 CHS, Los Angeles, CA 90095 USA; 3 Department of Emergency Medicine, David Geffen School of Medicine at UCLA, Los Angeles, CA USA; 4 Department of Medicine, David Geffen School of Medicine at UCLA, Los Angeles, CA USA; 5 David Geffen School of Medicine at UCLA, Los Angeles, CA USA; 6 Department of Health Systems Science, Kaiser Permanente Bernard J. Tyson School of Medicine, Pasadena, CA USA; 7 RAND Health, RAND Corporation, Santa Monica, CA USA

**Keywords:** Medicaid, Managed care, Access to care, Quality of health care, Continuity of patient care, Qualitative interviews, Academic medical centers, Health equity

## Abstract

**Background:**

Ambulatory access to academic medical centers (AMCs) for patients insured with Medi-Cal (i.e., Medicaid in California) is understudied, particularly among the 85% of beneficiaries enrolled in managed care plans. As more AMCs develop partnerships with these plans, data on patient experiences of access to care and quality are needed to guide patient-centered improvements in care delivery.

**Methods:**

The authors conducted semi-structured, qualitative interviews with Medi-Cal-insured patients with initial visits at a large, urban AMC during 2022. Participant recruitment was informed by a database of ambulatory Medi-Cal encounters. The interview guide covered Medi-Cal enrollment, scheduling, and visit experience. Interviews were transcribed and inductively coded, then organized into themes across four domains: access, affordability, patient-provider interactions, and continuity.

**Results:**

Twenty participant interviews were completed (55% female, 85% English speaking, 80% self-identified minority or “other” race, and 30% Hispanic or Latino) with primary and/or specialty care visits. Within the access domain, participants reported delays with Medi-Cal enrollment and access to specialist care or testing, though appointment scheduling was reported to be easy. Affordability concerns included out-of-pocket medical and parking costs, and missed income when patients or families skipped work to facilitate care coordination. Participants considered clear, bilateral communication with providers fundamental to positive patient-provider interactions. Some participants perceived discrimination by providers based on their insurance status. Participants valued continuity, but experienced frustration arising from frequent and unexpected health plan changes that disrupted care with their established AMC providers.

**Conclusions:**

The missions of AMCs typically focus on clinical care, education, research, and equity. However, reports from Medi-Cal insured patients receiving care at AMCs highlight their stress and confusion related to inconsistent provider access, uncompensated costs, variability in perceptions of quality, and fragmented care. Recommendations based upon patient-reported concerns suggest opportunities for AMC health system-level improvements that are compatible with AMC missions.

**Supplementary Information:**

The online version contains supplementary material available at 10.1186/s12913-024-11619-3.

## Background

Academic medical centers (AMCs) face renewed pressure to advance health equity, in addition to their long-standing commitments to clinical, educational, and research missions [1, 2]. One patient population that warrants prioritization in equity efforts includes patients insured with Medicaid (i.e., Medi-Cal in California). This cohort, often characterized by a greater share of racially and ethnically minoritized individuals [3], frequently resides near AMCs, but has often been overlooked as an economically marginalized group. By developing strategies informed by patient experiences to improve clinical care delivery across the spectrum of medical and social conditions, patient access could improve. Overcoming insurance barriers could decrease clinician burnout, increase patient satisfaction, and concurrently enhance the educational mission of the AMC by strengthening the knowledge base of its faculty and trainees [4]. 

In the United States, Medicaid serves as the largest health insurance program by enrollment [3]. In California, Medi-Cal (including the Children’s Health Insurance Program) is the source of health insurance for about one-third of residents [5], with over 85% of enrollees receiving health services through plans associated with managed care organizations (MCOs) [6]. MCOs receive upfront payments from states to deliver services to Medicaid-insured patients, though AMC participation in these networks varies. While AMCs often deliver a substantial portion of inpatient care to Medicaid-insured patients, they provide a smaller portion of ambulatory care to Medicaid-insured patients. Evidence on quality of care for Medicaid-insured patients within AMC ambulatory care is sparse [7–10]. 

Existing literature on quality of care for Medicaid-insured patients beyond AMCs points to several challenges despite gains following the passage of the Affordable Care Act. Survey data from Medi-Cal enrollees in California revealed persistent difficulty with timely appointments and other measures of access compared to employer-based insurance [11]. Evidence also suggests that Medicaid beneficiaries have experienced persistent racial and ethnic disparities in outcomes [3, 12, 13], less patient-centered care [14], and longer wait times [15, 16] compared to privately- and Medicare-insured patients. While informative, the majority of these studies rely on claims or survey data, which may lack contextual or nuanced information critical for improving patient-centered care.

Interviews with Medicaid beneficiaries give voice to a rarely heard group vulnerable to health inequities. Their reports may reveal unseen patient-provider issues impacting perceptions of care and unintended consequences of policy on individuals [17]. Research conducted in partnership with Medicaid-insured patients could also help prioritize concerns important to local community members [18, 19]. For example, studies exploring how Medicaid-insured patients choose plans may also inform useful strategies for MCOs that are developing patient communication strategies [20]. However, qualitative work on Medicaid patient experiences is currently limited to conditions, such as substance use disorders [21], contraceptive care [22, 23], and disabilities [17], or to policy assessments in specific geographies [20, 24, 25]. The present work helps fill these knowledge gaps by gathering qualitative experiences of a clinically diverse sample of Medi-Cal patients’ access and quality of care at a large, urban AMC.

## Methods

### Sampling, and participants

As part of a health system quality improvement project that followed health system changes in contracts with Medi-Cal managed care plans, we sought to learn from Medi-Cal insured patients about their experiences receiving care in an AMC. The protocol of this qualitative descriptive study [26] included a diverse sample across demographic characteristics including age, race, ethnicity, and preferred language of patients newly seeking care at a large, urban AMC.

Our three-step recruitment methodology began with data from a health system-generated list of Medi-Cal encounters of individuals with age (18–64), language (English or Spanish speaking), insurance (any Medi-Cal plan type except emergency-only Medi-Cal), visit type (any except behavioral health, which was excluded given the county’s special behavioral health insurance policies), and date of first scheduled AMC visit after January 1, 2022 (even if not attended to capture individuals with barriers to attending new visits). We specified the participants’ first visit at the AMC to be recent enough that interviews could be conducted while minimizing poor recall and telescoping, but delayed enough to capture follow-up experiences at the AMC. Second, we screened the medical record of each patient to verify the accuracy of participant’s demographic data as presented in the data log. Third, we contacted participants via telephone three times before they were deemed a “non-responder.” Given that this project was part of the AMCs quality improvement efforts, it was deemed exempt by the university Institutional Review Board.

### Data collection procedures

Semi-structured interviews were performed via telephone by experienced physician interviewers (MB, JF, DG, and VN) from 8/2022 to 6/2023. Using a prepared guide, interviewers asked open-ended questions, and probed emotional reactions to enrollment, scheduling, check-in at the AMC, and the clinical encounter itself (see Appendix 1 for complete interview guide). The guide was translated into Spanish with probes added to gather language-specific experiences, and subsequently back-translated to English to ensure accuracy by bilingual team members (DG, VN). Participants provided informed consent and received a $50 gift card for participation.

Interviews were audio recorded and transcribed in the participant’s spoken language using NVivo, then de-identified and reviewed for accuracy against the audio recording [27]. Spanish language transcriptions were translated to English by a bilingual team member and checked for content against the original audio to ensure subtleties of the interviews remained [28]. 

Physicians with training and experience in qualitative research used an inductive approach to code the qualitative data using NVivo software (MB, JF) [27]. After independently reading all transcripts, an initial set of four transcripts were coded together to develop the preliminary coding scheme, using concepts directly derived from transcripts. Discrepancies were reconciled through discussion. Codes that emerged later during the interview period were back-coded to earlier interviews as applicable. Investigators then independently coded the remainder of the interviews. The research team discussed emerging themes from the coded interviews and conducted code sorts to analyze the range of data across participants. We determined data saturation when the narratives and themes of the final interviews resulted in no new experiences. To describe a range of participant experiences and highlight narrative stories, we include vignettes that provide examples of our themes. We do not report frequencies of themes as these data were not intended to create generalizations [29]. 

Finally, we used a deductive approach to assign themes to broader domains of access and quality, stemming from the characterization of high-quality care as safe, effective, patient-centered, timely, efficient, and equitable [30]. The study team consolidated and redefined these into 4 unique domains that best reflected the salient findings in our analysis. Notably, continuity of care emerged as a distinct concept from our analysis. Throughout this manuscript, we refer to Medi-Cal-insured patients who completed interviews as “participants” rather than “patients” to focus on their lived experiences with care, including unique cultural values and expectations [31], and prioritize participants’ own subjective encounters with Medi-Cal and the AMC across varied health conditions [32]. Below we define the four domains:

- *Access*: Experiences securing insurance coverage, initial access to the AMC, timeliness of care, degree of physical, cultural, and linguistic accessibility of visit [33].
- *Affordability*: Monetary and non-monetary costs (e.g. time) to patients, family, and the health system [34].
- *Patient-provider interaction*: Patient-centeredness of communication, perceptions regarding equity of treatment, and contributors to trusting relationships [35–37].
- *Continuity*: Ability to continue care within the AMC, experiences of the coordination of clinical services [38], understanding how to obtain recommended follow-up interventions (diagnostics, results, treatments options), and guidance about clinical follow-up.

## Results

### Participant characteristics

Of 176 patients screened, 45 met eligibility criteria and were contacted; 27 patients answered the recruitment phone call and 20 completed the interview. Mean time from first AMC visit to the interview was 5.2 months and mean interview duration was 30 min. A slight majority (55%) were female, and most (85%) were English-speaking. Most (80%) self-identified as a minority or “other” race and 30% identified as Hispanic or Latino ethnicity (see Table 1). The majority (65%) were assigned to a Medi-Cal MCO during their care. Participants visited a range of clinical disciplines across primary care (40%) and medical or surgical specialty care (60%). For each of the four domains, the text that follows introduces themes and presents one clinical scenario.

**Table 1:** Characteristics of 20 participants

Characteristic	*N* (%)
Age, mean (SD, range)	35.4 (13.0, 18–61)
Sex, female	11 (55%)
Primary language	
English	17 (85%)
Spanish	3 (15%)
Race	
Alaska Native or American Indian	1 (5%)
African	1 (5%)
African American	3 (15%)
Armenian	1 (5%)
Indian (India)	1 (5%)
Japanese	1 (5%)
Multiracial	1 (5%)
Other/Not Listed/Unknown	7 (35%)
White	4 (20%)
Ethnicity	
Hispanic or Latino	6 (30%)
Not Hispanic or Latino	13 (65%)
Unknown	1 (5%)
Medi-Cal Plan	
Fee-for-Service	7 (35%)
Managed Care	13 (65%)
Visit Specialty	
Primary Care	8 (40%)
Medical Specialty	3 (15%)
Surgical Specialty	6 (30%)
Obstetrics/Gynecology	1 (5%)
Medical and Surgical Specialties	2 (10%)

*Abbreviation*: *SD* standard deviation

### Access

#### Theme 1: patient frustration stems from unfamiliar enrollment and MCO plan selection procedures

The majority of participants were new to Medi-Cal. For some, enrollment was uneventful with prompt coverage. Those who were assisted by patient navigators tended to encounter the fewest roadblocks, or were less distressed by them. Prolonged enrollment was associated with initial denials, the need to correct personal information, or difficulty entering a preferred MCO plan (see Table 2).

**Table 2:** Access domain themes and quotations from qualitative interviews

**Access Theme 1: Patient frustration stems from unfamiliar enrollment and MCO plan selection procedures**	
*Sub-Theme*	*Representative Quote(s) (Participant No.)*
Medi-Cal enrollment experiences varied from easy to prolonged	[Enrollment] was a nightmare, an absolute nightmare. I finally got on Medi-Cal, called up, […] spent almost an hour talking. Except that when I went to go to the doctor, [the clinic] called the day before I was going to go and said that I wasn’t in the right program [14].
Notifications of plan assignment from Medi-Cal MCOs often failed to reach participants	That switch from Medi-Cal [fee-for-service] to [an MCO] was unnoticeable. They don’t call you or tell you. My surgeon said, “you now have [an MCO].” I’m going to have to find another surgeon for the follow ups [5]
	[The office staff] called me to tell me that she was sorry, but they had canceled my appointment because of a change in insurance. And she gave me the information for who to call. And when I called them, they never answered. So that’s why I told her to cancel my [AMC] appointment. That’s how I found out [my insurance changed] [19].
Inability to access the AMC with certain MCOs stressed participants	They switched me from [one MCO] to something else and then something else to something else. Every time I tried to go back to my AMC, they said “we can’t provide you care.” […] It was just frustrating. I felt like Medi-Cal is supposed to be for [people with] low incomes, it’s supposed to take stress away, but instead it is now inducing stress [17].
**Access Theme 2: Health system structures pose challenges to receipt of timely care**	
*Sub-Theme*	*Representative Quote(s) (Participant No.)*
Appointment wait times caused distress, particularly when unmanaged symptoms persist	Yeah, yeah, it’s difficult to get an appointment. A few months [to wait for an appointment], yeah. [This delay impacts me] greatly. Very, very, very greatly. My issues impact my normal daily ability to even sit in class, and as someone who has a school schedule three days a week from eight a.m. to nine p.m. that doesn’t work for me, especially since I’m going to continue working [10].
Participants perceived delays in care that they attributed to unfamiliar health system processes	No, they didn’t refer me to anybody. They just said go find a family doctor, call one that takes your insurance and go see him, which is kind of ridiculous because they should have just taken an MRI of my knee and my ankle there […] I ended up having a broken toe and I end up getting an MRI a month later and I had a torn ankle [7].
**Access Theme 3: AMC staff composition and communication portals pose barriers**	
*Sub-Theme*	*Representative Quote(s) (Participant No.)*
English-only patient portals increased patient reliance on others to interact with health system	At that time, as my partner spoke English, He translated [the English patient portal text to Spanish] for me. But […] he goes to work and comes back late [19].

*Abbreviations*: *AMC* academic medical center, *MCO* Medi-Cal managed care organization, *MRI* magnetic resonance imaging

Many Medi-Cal enrollees reported the available documentation from Medi-Cal did not meaningfully guide them how to select among plans. Material was not readily accessible and participants were distressed after being assigned to plans that were far from home or did not include the providers they perceived to match their needs. Some participants did not realize they would be assigned to an MCO, and first learned of MCO plan assignments during a visit with a staff member at their AMC visit.

#### Theme 2: health system structures pose challenges to receipt of timely care

Most participants were able to easily schedule an initial appointment at the AMC through its call center. However, the duration from scheduling call to the clinical appointment varied, with longer wait times noted for some specialists. Among participants with long wait times, many expressed understanding, but participants with persistent, unmanaged symptoms were distressed (Table 2).

#### Theme 3: AMC staff composition and communication portals pose barriers

Among the three Spanish-speaking participants, family members or bilingual office staff often assisted in arranging the appointment. Participants felt the provision of telephonic or video interpreters was effective, but the English-only patient portal was a barrier to self-managing care (Table 2).

#### Access vignette

Navigating between Medi-Cal and AMC systems posed additional challenges for participants, as illustrated in the following vignette of a woman seeking midwifery care.

She spoke to an insurance specialist at the AMC in an attempt to enroll in one of the AMC’s contracted MCOs. This involved “a lot of following up, and it was a lot of challenges” navigating between the AMC and Medi-Cal’s MCOs. Despite her attentiveness to both the AMC’s and MCO’s suggestions, the MCO ultimately advised her that her MCO was not contracted with the AMC where she had scheduled a prenatal appointment. She recounted “between all that time, my pregnancy was progressing and I didn’t have any care.” Ultimately, she had to switch to a different health system that was contracted with her MCO. She reflected, “I must have Googled like a million things to try to figure this out, and it was just so hard. Like, okay, what plan specifically is covered by Medi-Cal for [the AMC]? It was almost impossible to figure out. And I’m a college grad and realize that if I can’t do it, I can’t imagine anybody else trying to maneuver it.” (Participant 6).

### Affordability

#### Theme 4: participants faced unexpected non-medical and medical costs

While direct healthcare costs were covered for participants, a minority cited ancillary costs such as parking and medications as burdensome, particularly when they were recurrent (see Table 3).

**Table 3:** Affordability domain themes and quotations from qualitative interviews

**Affordability Theme 4: Participants faced unexpected non-medical and medical costs**	
*Sub-Theme*	*Representative Quote(s) (Participant No.)*
Participants describe parking costs as a significant, but nonnegotiable burden	Well, [$15 parking] affects me. But I have to. [Paying is] better than getting a ticket. […] Yes, it’s difficult for me, but it has to be done. Because if not, how am I going to get to my appointment? [20]
Participants experience occasional unexpected out of pocket costs for care	A hundred and something dollars just for some of the vitamins that I need. Like I have to have them […] it has to be out of pocket [12].
**Affordability Theme 5: Support networks contributed uncompensated time to patient care**	
*Sub-Theme*	*Representative Quote(s) (Participant No.)*
Family members contribute time to care coordination and transportation	No, I had to call, make appointments, call different numbers, different places to see if they take my insurance and my mom helped with all that [7].
	My dad would actually take me. So he was working, but he would take a day off every time I had a doctor appointment and I couldn’t drive, and then my sister would go with me as well [2].
AMC staff, family and patients spend time calling Medi-Cal to obtain care	Obviously, a phone call to Medi-Cal is required and they have these insane wait times. And as someone who struggles with anxiety, I would spend four hours on the phone with these people and none of them were able to first of all get me the answer that I needed. And second of all, they kept giving me the wrong information […] Not everyone has four hours to spare, to make just one phone call where someone doesn’t even have the correct information for them [10].
	I definitely tried to [talk to Medi-Cal]. That doesn’t mean I got anyone. I was either hung up on after waiting for extensive periods of time or I was told that it was closed. I don’t think I ever talked to someone in the public program, ever. […] I had probably tried to call a total like eight times. Total waiting time was like probably four or five hours [17].

*Abbreviation*: *AMC* academic medical center

#### Theme 5: support networks contributed uncompensated time to patient care

Participants and their families forfeited needed employment hours and associated wages to make time to contact multiple providers or agencies, clarify insurance plan information, and arrange visits. Many participants relied on family members and friends to assure adequate care coordination, record keeping, and transport.

#### Affordability vignette

The following case revealed the costs that participants and families absorb in order to implement the specialized care recommended by the AMC.

One participant undergoing liver transplant evaluation described the ways in which the frequent visits (e.g., $15 for parking) amplified out of pocket costs which “start to add up.” Additionally, specific vitamins were “a hundred and something dollars” and not covered by Medi-Cal. He also reported a “family member is taking care of me instead of working. So, it becomes a financial thing when you don’t have a steady stream of income.” Despite these challenges, he spoke positively about his care and was grateful for the services he received. (Participant 12)

### Patient-provider interactions

#### Theme 6: feeling heard by providers was important to participants

Participants expected, and many received, timely, high-quality care from the AMC. Participants appreciated clear explanations, attentiveness, and responsiveness from their clinicians. However, some participants were frustrated, perceiving their concerns to be discounted and unaddressed. One participant, recognizing missed opportunities to ask the doctor questions, blamed himself for not voicing his needs (see Table 4).

**Table 4:** Patient-provider interaction domain themes and quotations from qualitative interviews

**Patient-provider interaction Theme 6: Feeling heard by providers was important to participants**	
*Sub-Theme*	*Representative Quote(s) (Participant No.)*
Participants trust providers less when conflicting agendas arise	We were there for a particular reason. The doctor was very much more concerned about giving vaccinations, some that weren’t needed. And so that was displeasing. I feel the issue that we were there for was not addressed in a very timely fashion, allowing it to progress and get worse before treating [4].
Time pressures on providers elicit disappointment when patient concerns are unexplored	I was telling [the doctor] how I was feeling my ankle was torn. And [they said], “no, I think you’re fine…you’re walking.” And, you know, I had a broken toe and a torn ankle and stuff, and they didn’t give me anything that I needed and the referral didn’t help. And it was just a headache. (Later in interview, referring to specialist visit: ) I wish that he told me a bit more about where it was torn, what was torn, stuff like that. I should have asked him more but it seemed like he was in a hurry [7].
Clear and accessible communication with providers encourage trusting relationships for participants	Oh, again very smooth, the doctor walked in I just told him everything that bothers me. And he gave two referrals, numbers I should call. And then he said, “see you in about a year to see how everything goes.” [5]
	If I have questions, I have access to [the patient portal], so I’m able to communicate with [my providers] that way, and so is my caretaker. And they’re really good with their responses. They always help try to brighten my day if I’m not feeling well or, things like that. They’re very personable and they’re very knowledgeable [12].
	I really liked my doctor. It’s very rare that they listen to you. I had a concern for my ovary that I’ve had previously and it actually almost killed me. [The doctor] listened to my concerns […] and she validated my concern, and gave me the ultrasound and the different things that I needed for the test to determine what needed to be done [13].
**Patient-provider interaction Theme 7: Health system interactions contributed to perceived insurance-based discrimination**	
*Sub-Theme*	*Representative Quote(s) (Participant No.)*
Prior perceptions of insurance bias influence how patients view subsequent health systems	[Offices are] typically really rude. And I think that it’s partly because […] they feel like, oh, you’re on Medi-Cal so you shouldn’t be asking questions. You should be happy to just get what you’re getting.” […] I wanted to have the doctor’s name so I can check out some reviews and they didn’t want to give me the doctor’s name. So basically, I made an appointment with I don’t even know who. [11]
Participants suspect differential treatment when providers discuss Medi-Cal coverage of clinical care	I really do feel like [insurance] was part of his decision on why he [the doctor] didn’t want to go through the process of referring me, because he was like, “Medi-Cal doesn’t really want to pay for that” […] I’m not less of a person because I have state insurance.” [13]
**Patient-provider interaction Theme 8: Providers discounting participant symptoms strained relationships**	
*Sub-Theme*	*Representative Quote(s) (Participant No.)*
Distrust of providers increase when shared decision making is lacking	I was really disappointed […] and I just felt like my experience with my own body and what works for me wasn’t really being taken into account [by the doctor]. I was back on the same medication, and I didn’t feel like I really have a choice around that [10].

*Abbreviation*: *AMC* academic medical center

#### Theme 7: health system interactions contributed to perceived insurance-based discrimination

A subset of participants reported that healthcare experiences at AMCs prompted perceptions of receiving suboptimal treatment due to their having Medi-Cal insurance. Distrust intensified when the AMC to which the patient was referred did not accept their MCO plan (Table 4).

#### Theme 8: providers discounting participant symptoms strained relationships

Some participants perceived receiving substandard care and wondered whether this was attributable to their having public insurance (Table 4).

#### Patient-provider interactions vignette

In the following vignette, the participant recalled his care for an ocular injury that left him with distrust of the provider and health system.

During an emergency department visit, a foreign object was removed from his eye and eye drops were administered. He felt the treatment worsened his vision and wondered if a mistake had been made regarding the administered eye drops. During follow up, he was advised to see a cornea specialist. However, he later learned the recommended specialist would not be covered by Medi-Cal, leaving him frustrated and distrustful: “I don’t know why a doctor would tell me that I need to get something from somewhere else where it’s not covered. […] Are you playing games? Are you withholding something?” He wondered whether his treatment and clinical complication had been managed differently due to his having public, rather than private, health insurance. (Participant 11)

### Continuity

#### Theme 9: participants preferred consistency in providers and health systems

Great effort was often made by participants to establish and maintain continuity within the AMC, though success was often outside their control. In some cases, new MCO plans assigned to participants were not contracted with the AMC, and patients were told they could no longer be seen at the AMC. This led to frustration and stress for many, since this required identification of new providers in their networks and often delayed recommended medical care. In a few cases, participants blamed the physician or the AMC for the disruption in care, without recognizing the role managed care physician networks played in the plan assignments (see Table 5).

**Table 5:** Continuity domain themes and quotations from qualitative interviews

**Continuity Theme 9: Participants preferred consistency in providers and health systems**	
*Sub-Theme*	*Representative Quote(s) (Participant No.)*
Participants value consistency in care by the providers with whom they’ve established relationships	We wanted to keep going to [the AMC] because that is where all my surgeons were. […] And so, until I was done seeing the [AMC surgeons], I wanted to keep going to there. Then once I was done with [the AMC], that’s when I switched over to [an MCO] [16].
	I preferred going to the main [AMC] just because I already knew some of the people. Not that I don’t trust the other [AMC sites] that would have probably been closer to me. Just when you’ve already gone through your deal with a team, […] they already know where you stand. They already know what you’re probably going to need [2].
MCO changes mid-care place patients in new networks that may exclude AMCs, leading patients to distrust the AMC’s commitment to their care	There was no communication at all. Like honestly, I’m just very disappointed in this doctor because in the end they had given me this medication that had me in excruciating pain. And when I called her to inform her, she suggested that I take more of that particular medication. And then [the symptoms] had me on bed rest and literally bleeding for 12 days. And I had something protruding from my uterus area. And when I called, I had to leave a message with her nurse. Then I called back, and all of a sudden, she was no longer my doctor and they were pushing me over to [a county hospital] and I thought that was very negligent of them [9].
Participants seek opportunities to change health plans to maintain care within AMC	There’s straight [fee-for-service] Medi-Cal where you could get seen anywhere and they’ll cover as long as they take Medi-Cal or there is Medi-Cal where you are assigned to a particular hospital. Once [I confirmed I switched to fee-for-service] I was able to get my care. […] Personally, for me, [the transition to fee-for-service Medi-Cal] took a week because my social worker kept calling and calling [15].
**Continuity Theme 10: Participants experienced fragmented services**,** though care coordination strategies**,** when available**,** improved experiences**	
*Sub-Theme*	*Representative Quote(s) (Participant No.)*
Participants endure inefficiencies accessing ordered imaging studies when Medi-Cal coverage limits settings in which imaging tests can be performed	I was not able to get X-rays at [my AMC] because they weren’t covered by Medi-Cal, even though they were ordered by my provider at my [AMC]. Apparently, […] certain offices don’t provide the same services to the same insurance as primary care. […] I had to just make a whole day out of going out to a different location to get my X-rays done [17].
	I got the MRI done, but I needed an X-ray as well. […] So it was like another headache where I had to go to another office and make another appointment to do all these other things instead of just doing it at the same office or the same place [as where I had the MRI]. It’s just a really frustrating situation [7].
Care coordination improves experience in and perception of AMC	I get brought into the loop with my caretaker and my coordinator after they’ve already discussed [my appointments]. I come in and they let me listen in and explain to me what’s going to happen [12]
	[The nurse navigator] helped me out. She told me, “I need you to reach out to these people. You will fill out these papers.” She also got approval from [AMC] for them to actually approve the visit because my Medi-Cal said it was inactive [2].

*Abbreviations*: *AMC* academic medical center, *MCO* Medi-Cal managed care organization, *MRI* magnetic resonance imaging

A small minority of participants in this position sought to re-establish their fee-for-service coverage, either due to their own health system knowledge or at the urging of social workers. Participants found advice helpful when received from family, care coordinators, or social workers on navigating this bureaucracy, and participants expressed gratitude that they were able to continue care at the AMC with their existing providers.

#### Theme 10: participants experienced fragmented services, though care coordination strategies, when available, improved experiences

Participants were often not advised that the ancillary service recommended to them may require a special authorization. Such advice was most likely to be needed if the MCO with which they were contracted did not cover the services they required (e.g., a particular type of imaging study) at their local AMC site. Without awareness of the need for a special authorization, participants often were distressed to learn that their needed test was delayed or missed. Furthermore, patients were sometimes billed for exams they felt should have been paid by insurance. The inherent complexity of large health systems, with multiple sites for ancillary services like imaging (some covered by their insurance and some not), was often difficult to navigate (Table 5).

Care coordination services eased this challenge and improved the patient’s experience with the AMC. Participants in specific clinical programs, such as trauma, liver transplant, and oncology were more likely than others to receive case management from AMC staff.

#### Continuity vignette

The vignette below describes how one participant experienced care that left her feeling abandoned by her care team.

A participant who recently established primary care at the AMC newly developed significant vaginal bleeding. This prompted a visit to the AMC’s emergency department, where she received iron infusions and oral medications. When her bleeding persisted, she called her primary care office, who advised her that she had been assigned to an MCO, and the AMC “no longer took her insurance.” After being directed to visit a county hospital for care, this patient reported feeling that the AMC was “judging [her] on the type of insurance [she] had”. She described feeling significant distress and felt the physician was “negligent” for referring her to a different institution. (Participant 9)

## Discussion

Through qualitative interviews with Medi-Cal insured patients, we explored health system perceptions across four domains of quality including access, affordability, patient-provider interactions, and continuity [30]. These domains readily emerged as central themes during our data analysis. We found that the complexity of care delivery for Medi-Cal insured patients at the AMC often created obstacles to participants’ care *access*. While participants had high regard for the care at the AMC, they also reported *affordability* concerns, including unexpected costs and family support required for participants to fully engage in care. Participants felt providers delivered the highest quality care when their autonomy was respected and communication was clear; whereas, when they had unaddressed concerns or providers referenced coverage limitations, they felt distrust within their *patient-provider interactions*. Lastly, *continuity* within the system was highly valued, but often not experienced. Altogether, this work highlights opportunities for AMCs to improve care delivery across ambulatory care disciplines for economically marginalized patients with Medi-Cal insurance.

While many studies show that access to care improves for those enrolled in Medicaid compared with the uninsured [39, 40], AMCs could benefit from further understanding of patient experiences and challenges navigating care to improve processes. While our participants revealed many positive enrollment experiences, the interviews also revealed negative emotional impacts of navigating nuances of Medi-Cal. Some participants also received conflicting or incomplete information from the AMC, the state Medi-Cal office, or the MCO regarding whether their plan networks would include the AMC. Fortunately, for many, health plan-related challenges often diminished with the passage of time and acclimation to the AMC and Medi-Cal program. This was especially notable among participants receiving care within clinical programs with embedded case management resources (i.e. trauma surgery, transplant, oncology), and for those receiving advice or support by social workers or nurse navigators.

To address insurance-related challenges, many participants employed their own time (often resulting in missed wages), self-advocacy, or family member’s time and resources to learn how to access and maintain their care within the AMC. Participants often described intense additional burden and stress involved in navigating the system, which was variably effective. Successful navigation was not consistently aligned with participants’ burden of illness, primary language, or demographics. Instead, successful navigation frequently depended on family or participant awareness of how the AMC *and* Medi-Cal systems administer care, and their understanding of the increasingly complex and fragmented US health system.

How well patients navigate complex US health systems is an area of increasing concern. Healthy People 2030 [41] defines health literacy to include the degree to which health organizations deliver understandable health information. They emphasize the importance of health organizations communicating clearly on the scope of health plan coverage and easing access to navigation services [42]. Our participants report experiences receiving care in organizations not consistently providing such clarity. Examples included receiving conflicting information between agencies, inability to clarify health plan coverage from the MCO or the AMC, and confusion when they were told they could no longer continue AMC care due to changes in their plan networks.

AMCs and Medi-Cal organizations share responsibility for proactively developing easier to navigate systems that use clear language that anyone can understand, regardless of their own health literacy. The goal of this concept, known as health literacy universal precautions, is to provide transparent, accurate, and accessible information to guide all patients, thus providing potential benefits to those challenged by health system complexities [41]. California’s state-level transformational efforts to enhance care management with additional coordination and community supports for high-need members across the state provides one model for this approach through California Advancing and Innovating Medi-Cal (CalAIM) [43, 44]. 

Given the multiple systems involved in delivering healthcare to Medi-Cal patients, solutions to improve patient navigation within Medi-Cal are also needed within individual AMCs and MCO health plans. Within AMCs, a team of case managers specifically designated to assist Medi-Cal-insured patients with access, quality, and social determinants of health concerns could leverage their experiences with diverse patients insured with Medi-Cal to systematically catalogue challenges and successful solutions. Next, MCO efforts to clearly specify the terms of their service contracts with transparent, comprehensive, and current material regarding disease-related, geographic, continuity, and fiscal concerns would allow AMCs and patients to make informed decisions about their care. This could be most effective if MCOs shared this material with both AMCs and patients to ensure streamlined, consistent information.

While historically AMCs have been known for their strength in delivering complex tertiary and quaternary care *inpatient* services, including to publicly insured individuals, access to comprehensive *ambulatory* care at AMCs is variable [7, 45]. Since AMC health systems commonly have a high density of specialists [46], Medicaid-insured patients with conditions requiring specialist consultation could benefit from process improvements between AMCs and MCOs, such as streamlined referral processing and health information sharing. Expanding partnerships between AMCs and Medicaid managed care can enhance the equity, education, diversity, and clinical missions of AMCs while improving patient access and outcomes. This has been suggested as a reason for AMCs to include more Medicaid ambulatory patients within their practices.

- From an equity perspective, Medicaid patients should be able to access comprehensive care and to benefit from continuity of care particularly within state-funded institutions such as AMCs [47, 48]. Innovation is needed to optimize care coordination for Medicaid-insured patients receiving primary and specialty care at AMCs and also for those referred from outside health systems to AMCs for episode-specific care [49]. New information technologies highlight opportunities for health systems to efficiently exchange administrative and clinical information across care settings while maintaining patient privacy [50].
- Considering the AMC educational mission, participants valued continuity of their care, including instances when trainees were often core members of the team. Resident trainees exposed to the system navigation, medical, and social challenges of their Medi-Cal-insured patients may enhance their motivation to participate in the Medicaid program in the future [51].
- As efforts to expand medical student diversity evolve, more trainees prioritize serving racially and ethnically minoritized patients, who are over-represented in the Medicaid population, during residency and beyond [52].
- Patients’ preferences for ambulatory AMC care should be respected, though this will require expansion of AMC ambulatory care services for patients receiving public assistance. Solutions for implementing AMC partnerships with MCOs may ultimately depend on the local health systems and patient populations that vary across Medicaid markets and geographies.

Our study within a single AMC sought to highlight how the AMC mission intersects with Medi-Cal patient needs. A strength of this analysis is its presentation of patient’s description of their lived experiences as Medi-Cal-insured patients seeking care within an AMC. Patient reports of concerns about care fragmentation, unanticipated costs, under and over referrals, and feeling unheard are not unique to Medicaid patients or unique to their receipt of care at an AMC. We highlight the voices of these patients because the literature consistently references challenges patients with Medicaid have maintaining consistent primary care coordination [53], accessing timely specialty services [54], and feeling their needs are being met with cultural humility [14]. Additionally, while AMCs traditionally were situated in urban centers in close geographic proximity to diverse populations, increasingly they have been expanding to more affluent areas [55]. As a consequence, individuals from communities with lower financial resources may have less exposure to AMCs and the mix of generalist and specialist opportunities they provide. Disparities in the delivery of health care services at AMCs can be mitigated when patients with Medicaid insurance, along with other insurance types, have the opportunity to receive care at AMCs. As a large academic center with a cohort of highly trained experts and trainees spanning disciplines, most AMCs offer the opportunity for diverse patients and provider teams to work together to address a plethora of medical and surgical conditions while also considering social and economic challenges associated with complex conditions.

Our findings build on existing understandings of affordability in healthcare. Our participants mainly relied on cars for appointment travel, and several mentioned repeated parking costs as distressing. To put $15 parking fees in context, patients earning the maximum individual income to qualify for Medi-Cal (138% of the federal poverty level) must pay nearly 4% of their weekly gross income for each visit, before accounting for mileage and other costs. Participants did not discuss alternatives such as public transportation or rides provided by insurers, though these options may be perceived as unsafe, time-consuming, or unreliable [56]. Further research could illuminate whether these alternative transit options are known among and available to Medicaid beneficiaries. In addition, while families serving as informal caregivers is acknowledged in the Medicaid literature [57, 58], this study uniquely shares the need for supports among younger, working Medicaid cohorts.

Our study has limitations. The recruitment was limited to English and Spanish speaking participants. Our findings reflect a single institution in a large city with a diverse healthcare market and our findings may not transfer to health systems in other geographies with different populations or Medicaid programs. Nevertheless, our study site may share commonalities with other AMCs, and adds to the literature describing factors influencing patients to seek AMC care [59]. Participants agreeing to be interviewed may be biased more negatively or positively than average Medi-Cal-insured patients, though our recruitment strategy was designed to minimize biases from referral or snowball sampling. Despite these limitations, we were able to recruit a sample that was varied across age, race, ethnicity, preferred spoken language, Medi-Cal plan, and clinical departments to maximize the range of experiences within the data.

## Conclusions

We explored the experiences of Medi-Cal patients seeking care at an AMC across several quality domains of access, affordability, patient-provider interactions, and continuity. We found that system-level complexity and insurance constraints were barriers to accessing care, as were unexpected costs and the need for family and social support in care navigation. While our participants relayed a positive regard and desire for care at the AMC, our analyses have revealed new opportunities to improve communication with providers and patient understanding of access limitations related to contracting between health systems. Altogether, this work highlights opportunities for AMCs to improve ambulatory care delivery for economically marginalized patients with Medi-Cal.

## Supplementary Information

Supplementary Material 1.

## Data Availability

The datasets used and/or analysed during the current study are available from the corresponding author on reasonable request.

## Abbreviations

AMC: Academic medical center
CalAIM: California Advancing and Innovating Medi-Cal
MCO: Medi-Cal managed care organization
MRI: Magnetic resonance imaging
SD: Standard deviation
