# Supplementary material for: Qualitative perspectives of Medicaid-insured patients on ambulatory care at an academic medical center: challenges and opportunities
Source: BMC Health Serv Res. 2024 Sep 27;24:1139. doi: 10.1186/s12913-024-11619-3 (PMC11428444; doi:10.1186/s12913-024-11619-3)
Supplement: Supplementary file 1 — Supplementary Material 1. [file 12913_2024_11619_MOESM1_ESM.docx]

**Online Appendix**

**Qualitative Perspectives of Medicaid-Insured Patients on Ambulatory Care at an Academic Medical Center: Challenges and Opportunities**

[Appendix 1: Interview Guide 2](#_Toc158634139)

# Supplemental Digital Appendix 1: Interview Guide

Patient Interview Guide (English)

“Thank you for agreeing to participate in this interview to help us understand and improve care delivery for patients with Medi-Cal insurance. We hope to learn from patients insured with Medi-Cal about what has worked well for you. We also hope you will share with us any difficulties or delays gaining access to what you believe you need for your health care.

In the next 20-30 minutes, we would like to ask you some questions to help us identify areas where UCLA [the academic medical center] can improve common issues that arise for patients with Medi-Cal insurance. We might ask about getting referrals all the way through scheduling of appointments and billing.

We would like to remind you that your participation in this conversation is voluntary with no penalty if you decline or choose not to answer any of the questions. Your participation will not impact your clinical care nor your relationship with your healthcare providers. If you agree to participate, we will ask you to describe your experience at UCLA including any barriers to care, and any suggestions you may have. This call will take about 30 minutes.

We will record the interview if you agree, Your identity and personal information will not be disclosed, and our notes will not have your name attached. Your answers are confidential and will not be shared with health care providers. These notes will be kept in a password-protected, secured location. You may contact us at the phone number or e-mail address provided in the recruitment letter if you have any questions.

Do you have any questions or would it be okay to begin?”

***MAIN QUESTIONS:***

1. Medi-Cal

I would like to confirm that during the visits you had at UCLA, you had Medi-Cal insurance

*[If yes]*

- When did you first get Medi-Cal?
- What was that process like?
- Are you aware if your Medi-Cal is through a certain health plan? (Examples include LA Care, Blue Cross)

1. Broad UCLA experience

Tell us about your experience getting your first appointment at UCLA.

- *[Prompt:]* How did you learn about the steps that you would need to schedule an appointment?
  - Get insurance authorization for the appointment?
  - Register for and complete the appointment?
- Did you feel like you were adequately advised about how to get these steps completed?
- Was there confusion?

Positive Points

I hope you had some good experiences at UCLA. Can you tell me about your best UCLA experience?

*[Prompts:]* What did you like about it? What did UCLA do right for you?

Challenges

Can we now go to the other end of the spectrum? Would you mind sharing with me at least one example of an experience you had getting care at UCLA that disappointed or frustrated you?

*[Prompts:]* Were there some aspects of your UCLA experiences that really upset you? Can you tell me what happened that upset you?

Did you have concerns that your insurance type was delaying or making it more difficult to get some of the care you hoped to receive?

**[IF SO]** Do you know why UCLA did not provide this service for you?

Had you previously received this service?

Do you understand why you did not receive it?

- Do you believe not receiving that service has harmed you?

***SOLUTIONS AND IDEAS:***

Our goal is to make the experience at UCLA accessible and responsive to you needs.

- *[Probe:]* What changes do you think we could make to remove barriers for patients with Medi-Cal insurance getting outpatient clinic care at UCLA?
- What would have made it easier for you to receive/access the care you want care at UCLA?

***ABOUT UCLA:***

Think back to before you tried to schedule an appointment. Why did you choose UCLA?

- What are your and your family’s perceptions of care at UCLA?

Can you be specific about at least one aspect of care received at UCLA that you had not received in other settings?

If you had options for where to see UCLA healthcare providers, would you prefer to see them at a satellite clinic in your community or would you prefer to come to one of the main UCLA facilities (Prompt if needed: for example, in Westwood)?

***CLOSING QUESTIONS:***

- Is there anything else you want for us to know that could help with making things better for patients like yourself with Medi-Cal?

***IF TIME:***

Financial Clearance

- Did you know that you needed to be financially cleared for your appointment after it was made?
- **[IF SO]** Were you aware of when you were financially cleared to attend your appointment?
- Did you need to talk to anyone outside of UCLA about your insurance? (For example, Medi-Cal, or LA Care)

Referral

- Were you ever referred to a specialist for care at UCLA?
- **[IF SO]** What type of specialist?
- Tell me about that experience.
- How long did it take to see the specialist after the referral was placed?

Clinical Encounter

- Tell us about your visit with the medical provider at UCLA
- Did you need to use an interpreter during your visit? If so, was there an interpreter readily available to you?
- How did you arrive to your appointment? Did you have any challenges getting there or parking?

Follow Up

- What happened after your appointment ended?
- Were you contacted again?
- Did you receive results of any lab tests or imaging?
- Did you schedule another appointment?

Patient Interview Guide (Spanish)

"Gracias por estar de acuerdo con participación en esta entrevista para ayudarnos a entender y mejorar la provisión de atención médica a los pacientes con seguro de Medi-Cal. Esperamos aprender de los pacientes asegurados con Medi-Cal sobre lo que ha funcionado bien para usted. También esperamos que comparta con nosotros cualquier dificultad o retraso en el acceso a lo que cree que necesita para su atención médica.

En los próximos 20-30 minutos, nos gustaría hacerle algunas preguntas para ayudarnos a identificar las áreas en las que el sistema de salud puede mejorar los problemas comunes que surgen para los pacientes con seguro de Medi-Cal. Podríamos preguntar sobre la obtención de referencias hasta la programación de citas y la facturación.

Nos gustaría recordarle que su participación en esta conversación es voluntaria y que no se le penalizará si usted rechaza o decide no responder a alguna de las preguntas. Su participación no afectará a su atención médica ni a su relación con sus proveedores de atención médica. Si está de acuerdo en participar, le pediremos que describa las formas en que interactúa con las necesidades de los pacientes de Medi-Cal, sus observaciones sobre las barreras para tener visitas clínicas en UCLA y cualquier sugerencia que pueda tener. Va a durar entre 20 y 30 minutos.

Su identidad e información personal no serán reveladas, y nuestras notas no tendrán su nombre. Sus respuestas son confidenciales y no serán compartidas con los proveedores de atención médica. Estas notas se guardarán en un lugar asegurado y protegido por contraseña. Puede ponerse en contacto con nosotros en el número de teléfono o en la dirección de correo electrónico indicados en la carta de reclutamiento si tiene alguna pregunta.

¿Tiene alguna pregunta o le parece bien empezar?"

**MAIN QUESTIONS:**

1. Broad UCLA experience

Cuéntenos su experiencia al conseguir su primer nombramiento en UCLA.

- *[Prompt:]* ¿Cómo se enteró de los pasos que debía seguir para programar una cita?

o ¿Obtener la autorización del seguro para la cita?

o ¿ Registrarse y completar la cita?

- ¿Cree que se le informó adecuadamente sobre cómo realizar estos pasos?

- ¿Hubo confusión?

1. Positive Points

Espero que haya tenido buenas experiencias en UCLA. ¿Puede contarme su mejor experiencia en UCLA?

- *[Prompt:]* ¿Qué le gustó? ¿Qué hizo bien UCLA para ti?

(3) Challenges

¿Podemos pasar ahora al otro extremo del espectro? ¿Podría compartir conmigo al menos un ejemplo de una experiencia de atención en UCLA que le haya decepcionado o frustrado?

- ¿Hubo algún aspecto de su experiencia en UCLA que le molestó mucho? ¿Puede decirme qué fue lo que le molestó?

- ¿Estaba preocupado que el tipo de seguro que tenía estaba retrasando o dificultando a recibir la atención lo que esperó?

- **[IF SO]** ¿Sabe por qué UCLA no le prestó este servicio?

- ¿Había recibido este servicio anteriormente?
- ¿Entiende por qué no lo recibió?
- ¿Cree que no recibir ese servicio le ha dañado?

**SOLUTIONS AND IDEAS:**

Nuestra meta es hacer que la experiencia en UCLA sea accesible y responda a sus necesidades.

**- *[Probe]* ¿Qué cambios cree que podríamos hacer para remover las barreras para que los pacientes con seguro de Medi-Cal reciban atención en las clínicas ambulatorias en UCLA?
- ¿Qué habría hecho más fácil para usted recibir/acceder a la atención que desea en UCLA?**

**ABOUT UCLA:**

Piense en lo que hizo antes de intentar de programar una cita. ¿Por qué eligió UCLA?

**- ¿Cuál es su percepción y la de su familia sobre la atención en UCLA?**

- ¿Puede especificar al menos un aspecto de la atención recibida en UCLA que no haya recibido en otros lugares?

**- ¿Si tenía opciones para donde ver proveedores de atención médica de UCLA, preferiría verle en una clínica en su comunidad o preferiría venir a una de las facilidades principales en UCLA (Prompt if needed: por ejemplo, en Westwood)?**

**FINAL QUESTIONS:**

- ¿Hay algo más que quiera que sepamos con que podría ayudar con mejorar las cosas para los pacientes como usted con Medi-Cal?

***IF TIME:***

Autorización financiera

- ¿Sabía usted que necesitaba autorización financiera para su cita después de haberla programada?
- [**IF SO**] ¿Sabía usted cuándo fue autorizado financieramente para asistir a su cita?
- ¿Usted tuvo que hablar con alguien fuera de UCLA sobre su seguro? (Por ejemplo, Medi-Cal o LA Care)

Referencia

- ¿Alguna vez fue referido a un especialista para ser atendido en UCLA?
- [**IF SO**] ¿Qué tipo de especialista?
- Cuénteme sobre esa experiencia.
- ¿Cuánto tiempo tardó en ver al especialista después de la referencia?

Encuentro clínico

- Cuéntenos sobre su visita con el proveedor médico en UCLA
- ¿Necesitó utilizar un intérprete durante su visita? Si es que si, ¿hubo un intérprete fácilmente disponible para usted?
- ¿Cómo llegó a su cita? ¿Tuvo algún problema para llegar o para estacionar?

Seguimiento

- ¿Qué ocurrió después que la cita acabo?
- ¿Se pusieron en contacto con usted de nuevo?
- ¿Recibió los resultados de alguna prueba de laboratorio o de imagen?
- ¿Programó otra cita?
